# What role conceptions do multi-healthcare professionals have of physicians and what role expectation do they have of physicians in a community?

**DOI:** 10.1186/s12875-021-01568-9

**Published:** 2021-11-02

**Authors:** Junji Haruta, Ryohei Goto, Sachiko Ozone, Tetsuhiro Maeno

**Affiliations:** 1 Medical Education Center, School of Medicine, Keio University, 35 Shinanomachi, Shinjuku ku, Tokyo, 160-8582 Japan; 2 Department of Primary Care and Medical Education, Faculty of Medicine, University of Tsukuba, Tsukuba, Japan; 3 Department of General Medicine and Primary Care, Faculty of Medicine, University of Tsukuba, Tsukuba, Japan

**Keywords:** Community medicine, Physicians, Interprofessional relations, Primary health care, Qualitative research

## Abstract

**Background:**

To create an effective community-based integrated care system, interprofessional collaboration based on healthcare professionals’ mutual understanding of their respective roles must be promoted. This study aimed to identify the role conception and role expectation that other healthcare professionals have towards physicians in the context of a community-based integrated care system.

**Methods:**

We organized focus groups and adopted ‘Role Theory’ as a theoretical framework. We collected data from healthcare professionals attending a conference on community-based integrated care systems in Japan. Fifty-four non-physician healthcare professionals consented to participate in 7 focus groups. Theme analysis based on the verbatim recorded transcripts was conducted in accordance with the framework of “Role Theory”.

**Results:**

The role conception of physicians is as a figure of intellectual authority positioned at the top of a traditional hierarchy, with a personal character of criticism/autonomy/closedness, not accommodative of interference from others, and upholding the Biomedical Model as an absolute standard. In response to this, the role expectation of physicians in the community is that they undertake actions that only physicians can undertake to ensure that a flat organization functions properly in providing medical explanations during patient transitions, and to offer healthcare support for patients who are difficult to access. This role expectation also includes the perception of patients as human beings, with physicians adapting to the Bio-Psycho-Social Model, explaining to patients about their disease as an authoritative voice based on an understanding of psychosocial circumstances, and sharing the prognosis of disease or disability. The expected personal character is a person with an open mind who allows others to seek advice, as well as a sense of approachableness which facilitates such seeking of advice.

**Conclusion:**

In the context of a community-based integrated care system, physicians should consider the understanding of their role conception and role expectation that other professionals have of them, and endeavor to create an open relationship with all healthcare professionals while giving careful consideration to their own role.

## Background

Many developed countries are experiencing an upsurge in healthcare needs because of the increase in the number of elderly people, who are more prone to multimorbidity and are affected by psychosocial factors [1]. To address this situation, healthcare professionals must not only strengthen their own professional point of view but also understand the views of others, and share information so that they can offer seamless services that meet patient needs [2]. In particular, the super-aging society has arrived in Japan, ahead of the rest of the world. The Japanese Government has announced its aim of creating a community-based integrated care system by 2025 that seamlessly links care and support in the community with the treatment of diseases [3]. To create such a system, interprofessional collaboration forged on an understanding of the roles of multi-professions is essential [4].

**Fig. 1:**
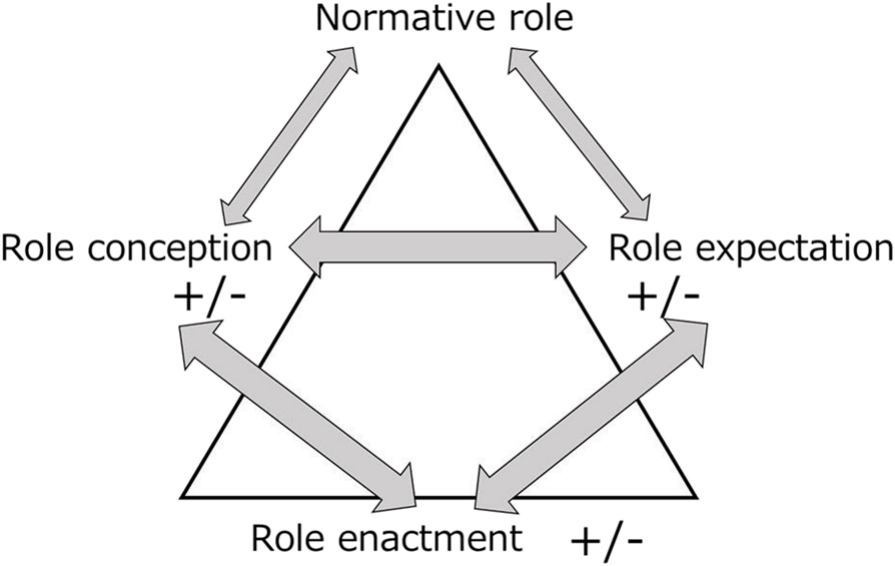
Relationships among role conception, role expectation, and role enactment

As a theoretical framework for the understanding of these roles, our study referred to ‘Role Theory’ [5]. The term ‘Role’ includes both exhibited behavior and expected behavior [6]. According to Role Theory, roles can be normative roles defined from an overall perspective or interpretative roles defined from a closer focus on individual subjects [5]. In role behavior occurring in a complex system, such as a community-based integrated care system, it is critical that the role of multi-professions be realistically understood not as a traditional normative role; rather, because the role is defined by continual interpretation of the social circumstances surrounding individuals, it must be understood in terms of mutual interpretations of actual roles by professionals in multi-professional teams. Figure 1 illustrates the concept of role theory by Morris [7], as revised by the authors. The presence of a minus sign between role concepts, role expectation, role enactment, and normative role can be interpreted to indicate some kind of role conflict.

Notably, in the context of the community, where issues that arise are diverse, role conception and role expectation, which are interpretative roles, are influential. This influence is in addition to that of the normative role, which is of a fixed nature [5]. Even if a physician has internalized the Bio-Psycho-Social Model as an interpretative role [8], other healthcare professionals may observe the physician’s exhibited behavior, and may find it impossible to build a flat relationship in the face of the physician’s uncompromising role conception based on the Biomedical Model. If discrepancies arise between the role conception and role expectation of physicians’ roles in a community setting, these roles may fall into a state called “role ambiguity” [9]. In mirroring this, other healthcare professionals then come to adopt a tacit role expectation without externalization. This is similarly manifested by medical students who will become doctors in the future, and who are confused by the conflict between idealism and realism in the professional reality they aspire to [10]. It is therefore required that physicians, as one of their interpretative roles, understand the discrepancies that exist between role conception - which is affected by exhibited behavior - and role expectation - which is affected by expected behavior. This is because physicians have to play the role of a hub between medical care based on a diagnosis and medical treatment within a community-based integrated care system.

Previous findings show that different healthcare professional hold different images of the interpretative role of physicians. Nurses working in clinical settings recognize the physician as playing the role of communicating the patient’s needs [11]. Pharmacists expect physicians in their partnership to enhance the pharmacists’ abilities and to exercise leadership in the clinical setting [12]. Psychiatric social workers expect physicians to not only provide patient care but also give guidance and support to the professional team. Because it is sometimes difficult to exchange information in a timely manner with specialist physicians, other healthcare professionals have reported bypassing general physicians and seeking advice directly from specialists [13]. This illustrates the fact that each healthcare profession has different expectations of physicians, and that physicians therefore need to understand the roles and responsibilities of different professionals and to give recognition to them [14]. Past findings have indicated that multi-professional relationship building encounters obstacles where there is no sharing of mutual expectations [15]. Despite this, knowledge about how different healthcare professions interpret community physicians is scarce.

We therefore considered that, by clearly identifying physicians’ role conception and role expectation, we could share how other healthcare professionals understand physicians. In the present context of a community-based integrated care system, the sharing of physicians’ interpretative role – which is evident in dynamic mutual-process action - will assist physicians in coming to understand their own roles and responsibilities.

In this study, we aimed to identify the role conception and role expectation of community physicians using focus groups (FGs) of healthcare professionals other than physicians.

## Methods

### Research methodology

We adopted the general inductive approach [16, 17]. A feature of this approach is methodological flexibility: it is not guided by any established qualitative methodologies, but is rather a systematic set of procedures for analyzing qualitative data which can provide reliable and valid findings. It aims to allow research findings to emerge from frequent, dominant, or significant themes inherent in the raw data in the context of focused evaluation questions. To meet this aim, thematic analysis of the data was conducted.

### Theoretical framework

This study is focused on the knowledge-cognitive aspects of role conception and role expectation based on Role Theory. We deliberately did not include “role enactment,” which is a cognition-behavior aspect. Although Role Theory considers that a normative role affects role conception and role expectation, and that the mutual interaction of factors with the addition of role enactment makes the roles apparent (Fig. 1) [7], role enactment is greatly influenced by individual behaviors based on their character as well as the history, culture and values of the community, which therefore makes it possible for local characteristics or ‘color’ to be overly represented. Accordingly, we focused here on the knowledge-cognitive aspects of multiple healthcare professionals by elucidating intersubjective facts.

### Research method

We adopted FGs, which are intended to elicit open-ended responses conveying thoughts or feelings, as the research design [18, 19]. FGs represent a qualitative data collection approach aimed at uncovering people’s thoughts and values [20]. However, unlike interviews, the researcher plays a peripheral role, acting as a “facilitator” [21]. We therefore adopted the FG approach as consistent with our aim of revealing the credible thoughts and feelings of the participants.

### Population and setting

We selected a community in X Prefecture, Japan. JH had worked in this community’s 30-bed public hospital, which provides outpatient services and home visits in the community, between 2015 and 2017. He established relationships with healthcare professionals in this hospital during this time, but no longer worked there. The community is located in a city with a population of 70,000 at the center of the prefecture. The community public hospital provides primary healthcare services that meet community needs, and also cooperates with an advanced treatment hospital located 10 min away by car, as well as other clinics and welfare institutions. In total, the community has around ten medical establishments, including a 36-department, 500-bed prefectural central hospital, a 100-bed hospital with a mix of acute and chronic/rehabilitation beds, a hospital that combines general wards and long-term care facilities, and outpatient-only clinics. In addition, the community also has five home nursing stations and fifty care managers working as healthcare professionals, who primarily support the at-home needs of patients/users within the community-based integrated care system.

### Data collection

FGs for healthcare professions in the community were held in January, 2019. The healthcare participants were divided into groups of seven and given 30 min to hold discussions. One of the authors (JH) presented the groups with prompting questions without joining the groups. The other authors (SO, RG, and TM) did not participate in the FGs; instead, staff working at the community general support center played the role of facilitator. Generally, a facilitator is someone who helps a group of people understand their common objectives and assists them to plan to achieve those goals without taking a particular position in the discussion [21]. JH asked the facilitators to closely listen to the participants’ opinions with the aim of finding the right moment to flexibly ask prompting questions. Prompting questions were developed collaboratively by JH, RG, and SO according to the research question and theoretical framework. Thus, the participants were asked main prompting questions on “what image they had of the physicians who they work with” and “what they expect of physicians in a community-based integrated care system”. The FGs were divided so that the participants were discrete in terms of main profession, sex, and affiliation. Since studies using semi-structured guides report that at least three to six focus groups are likely to identify 90% of the themes captured, we asked seven cooperating staff members to divide the group into seven FGs. Because the number of staff facilitators who cooperated in the research was limited to seven [22], seven groups consisting of 7–8 people each were established. FGs were conducted in a quiet room in a community center. All audio records of the FGs were transcribed verbatim, which the researchers commissioned to agencies that specialize in transcribing. Researchers took field notes while observing FGs from the outside and asked the cooperating staff what they noticed during the FGs with regard to non-verbal data [21]. Points noted by the researchers in their observation of participants and by the cooperating staff were also shared during the analysis [21].

JH, SO and TM are general practitioners, and RG is a physical therapist. JH has received training in qualitative research as part of a PhD program, while SO, RG, and TM received this training after obtaining their PhD degrees.

### Participants

In this local community, care conferences are held every 2 months to help attendees solve challenges they are facing or learn about new topics. Attendees are mainly welfare staff, with relatively few medical staff. The present study provided these professionals with the opportunity to exchange information with physicians working in the community hospital and clinics. To recruit participants, we informed previous care conference attendees in person or by postal mail that the next care conference 2 months hence would be held as part of the study. We also directly encouraged healthcare professionals who attended a care conference in January 2019 to take part in the study as a local accessibility-based convenience sample [23].

Participants comprised a wide variety of professions, including nurses and pharmacists, as well as administrative staff (including care workers and social workers), occupational therapists, physical therapists, care managers, and medical social workers. Administrative staff (including care workers and social workers) often propose community-wide policies and take care of patients who are welfare recipients or have mental disorders and who have severe care requirements. Care managers are responsible for planning care services provided under Japan’s long-term care insurance system [24]. Facility caregivers assist facility residents with activities such as meals, toileting, and bathing.

At the start of the FGs, they were briefed on the research being conducted and informed that they would be put at no disadvantage if they did not agree to participate. First, the *participants completed* a list of questions about their personal *background*, including sex and main profession in the community. They were then divided into seven FGs such that the professions and workplaces of participants in each group differed. The researchers did not participate in the FGs, but managed the whole project. One researcher (JH) contacted the participants via collaborating staff in the community general support center who managed the care conferences. These staff also maintained lists of individual FG participants and confirmed their details.

### Analysis

A verbatim record of each FG was analyzed by JH using theme analysis [25]. The validity of the findings was discussed with SO and RG based on “Role theory”. In accordance with the framework of “Role Theory” [6, 7, 9, 26], we focused on the thoughts and values concerning normative roles, and on the exhibited behaviors of physicians witnessed by the participants which influenced their role conception and role expectations of doctor-patient and physicians-other professional relationships. This allowed the identified themes to then emerge consistently across the series of data derived from the FGs. The main focus was on the analysis of verbal data, but observational data obtained by researchers and facilitators were also used as complementary explanatory material. TM then confirmed the consistency of the presented data and the findings, as well as the transferability of the findings. All researchers finally reviewed the data, including the appropriateness of sampling, and critically examined the themes to ensure the robustness of the data and analyses [27]. To ensure the validity of the analysis findings, member checking [28] was conducted among multi-profession participants who attended a care conference in April, 2019. There, JH shared the emerged themes of role conception and role expectation that the researchers had analyzed, and the verbal data associated with these themes. If there was any disagreement or doubt about the analysis, participants were also able to provide opinions through the collaborating staff [28].

### Ethical approval

This study was reviewed and approved by the research ethics committee of the University of Tsukuba (No 1353–1). All study participants provided informed consent prior to participation. To protect the anonymity of the participants, quotes in this paper are identified by randomly assigned professions and alphabet codes rather than participant names or initials.

## Results

### Participants

Of the 55 participants, one participant did not consent to taking part in the study.

Data from this participant’s group was therefore excluded from the analysis. The consenting participants were 18 males and 37 females, consisting of 15 care managers, 9 administrators, 6 occupational therapists, 4 physical therapists, 3 pharmacists, facility caregivers, and MSWs each, 2 nurses, and 10 other professionals.

### Findings

#### Role conception

Three themes emerged through the analysis of role conception: 1) Traditional hierarchy, 2) Physician-centered biomedical model; and 3) Personal character of criticism/autonomy/closedness. Two or three sub-themes were sampled for each theme (Table 1).

**Table 1:** Theme Analysis for role conception

Theme	Sub-theme	Example of text
Traditional hierarchy	Paternalistic intellectual authority	*A pharmacist said, “Should we consult with a physician directly about such a casual thing?”*
	Unreachable and of superior rank in hierarchy	*B care manger said, “We have the preconception and strong notion that because they are physicians, we have to be careful in the way we communicate.”* *C care manger said, “How can we speak to physicians tactfully so that they would ‘come down’ to our level?”*
	Unchallengeable authority	*D care giver said, “Simply because they are physicians, we tend to stand on guard.”*
Physician- centered Biomedical Model	Adherence to physician’s diagnosis of disease, excluding all else	*E government staff said, “We hear from patients that they were only able to tell the physician a few things when they come face to face.”*
	Absolute value of recovery from disease	*F MSW said, “It’s about individual words that are used but we want physicians to make a clearer distinction between the positive and negative (when explaining about the disease). We don’t know what to do when physicians tell us what not to do (in order to get better from an illness).”*
Personal Character	Criticism	*G MSW said, “We can’t say anything because we would be in trouble if someone says ‘the care manager said so without consultation.’”*
	Autonomy	*H care manager said, “There are physicians who do not see patients even if patients request it.”*
	Closedness	*I care manager said, “I don’t want us to be apportioning blame to each other but we find it difficult when we are told caregiving is for you to do and dispensing drugs is for us to do, and so on.”* *J Daily-life Support Coordinator said, “Do all physicians get notified by letter when a community-based integrated care conference is held? We never get physicians to attend.”*

#### Traditional hierarchy

Physicians are perceived as figures with paternalistic intellectual authority, and some participants had negative feelings about them. Meanwhile, some perceive physicians to be at the top of a hierarchy - figures who are out of their reach and with whom they do not share a common language or platform, and with whom they therefore cannot engage in discussion on an equal level and on the same plane. Physicians were also regarded as figures of authority who cannot be challenged.

*A MSW said, “I was told to go into a care facility because I cannot live alone at home, in a top-down attitude, or I was told not to go out on my own anymore.”*

*A care manager said, “It is difficult to talk with physicians, but even if I want to listen to the issues of patients on the phone, it does not happen.”*

#### Physician-centered biomedical model

Physicians seem to have confidence in their control of the Biomedical Model which other professionals do not possess. Based on this confidence, they believe in the absoluteness of their own diagnoses and exclude all other interests, leading sometimes to their disregarding of concerns raised by patients or other professionals. When these experiences are repeated, patients and other professionals find it impossible to ask physicians about diseases. They had also developed a mental image of physicians behaving in such a way as to give absolute credence to treatment/recovery criteria based on the Biomedical Model, and to disregard factors that exacerbate the disease.

*A MSW said, “I think it's common for physicians in hospitals to ask only about disease.”*

*An occupational therapist said, “We cannot speak out (even if we have doubts) against physicians’ diagnosis and such things, so we just listen.” (some physical therapists agreed)*

#### Personal character of criticism/autonomy/closedness

Physicians sometimes seem to exercise the autonomous decision-making they possess as specialist professionals in a powerful way, such as by refusing to take patient wishes into consideration and engaging in healthcare activities that are unfamiliar to other professionals but without consulting them. They sometimes take an attitude of criticism if their autonomy is infringed. Such actions by physicians have created issues for other professionals. An image of closedness has emerged, because physicians and healthcare professionals are not proactive in building partnerships among themselves.

*A Daily-life Support Coordinator said, “Physicians running private clinics don’t have networks with other practitioners.”*

*A pharmacist said, “Physicians sometimes prescribe drugs without sufficient knowledge (about new drugs), which creates problems for us.”*

## 2. Role expectation

Similarly, three themes were explored for role expectation: 1) A community team member in a flat organization; 2) Bio-Psycho-Social Model; and 3) Personal character of open-mindedness and approachableness that facilitates requests for advice. Two to three sub-themes were sampled for each theme (Table 2).

**Table 2:** Theme analysis for role expectation

Theme	Sub-theme	Example of text
Flat organization	Smooth coordination/referral between physicians	*K care manager said, “In the early stages, it’s all right just to see patients in ordinary clinics but when specialist treatment becomes necessary, we’d like physicians to make referrals to other physicians.”*
	Transitional support in the healthcare frontline (Transition)	*L MSW said, “If physicians can give specific advice, I think families would find it easier to accept when I talk to them. For example, in caregiving, if physicians can tell them that in such a situation, they could still cope at home using a particular method, or that there is this good way of doing things, and so on.”* *M care manager said, “When patients move to new hospitals, there are some things that care managers cannot explain, such as things related to our role in the new hospital or coordination with the patient’s attending physician, so we would like physicians to give a medical briefing to the staff at the new hospital at the time the patient is transferred. There are patients who don’t fully understand that there are changes in roles.”*
	Support that connects to healthcare (Connect)	*N care manager said, “If physicians can become part of the healthcare team in the case of patients who cannot connect to welfare services or healthcare due to refusal, and if they can then connect them to healthcare, would it be one form of support?”*
Bio-Psycho-Social Model	Attitude of treating patients as individual persons	*O care manager said, “I’d like to see physicians adopt the role of not just dealing with the disease but of adding enjoyment or meaning to patients’ lives. Often patients are asked only about their illness when visiting hospitals, but it would be good if they could also be asked about what gives them purpose in life or about their daily activities.”*
	Explanation from someone of authority in medical science	*P care manager said, “I’d like to see physicians say things more sternly to patients (even those with psycho-social issues) when necessary. This will enable co-medicals to give them support in their daily lives.*
	Explanation about diseases that affect their daily lives and about the paths to recovery	*Q MSW said, “In particular, physicians should teach patients or family how to conduct rehabilitation and take care of the patient at home. I think families will easily accept such teaching by physicians and commit to do them at home.”*
Personal Character	Open mind	*R pharmacist said, “There are 13 or 14 drugs that are similar – as specialists on medicines, we can explain these things, (…*) *so we really want physicians to listen to our advice.”*
	Approachableness	*S care manager said, “We understand that physicians are busy but we’d be glad if they could be easily accessible for consultation either by phone or fax.”*

### A community team member in a flat organization

Regarding the role of team members in the community, other healthcare professionals expect physicians to play a role that only physicians can play. With regard to issues relating to disease, they expect physicians to coordinate with each other if they encounter difficulties in diagnosis or treatment. They expect physicians to provide explanations as medical experts about transition to different healthcare locations and the scope of at-home healthcare. They also expect physicians to actively intervene for patients who do not have access to healthcare despite having an existing healthcare need.

*A nurse said, “We’d appreciate it if physician-to-physician, they can adopt the style and approach that they are ‘treating a particular patient.”*

*A care manager said, “I would like physicians to give me a medical explanation at the time of transfer to the community or home.”*

### Bio-psycho-social model

There was a Role Expectation for physicians not only to use a reductionistic Biomedical Model for patients but to treat patients as human beings in a Bio-Psycho-Social perspective, as multi-faceted and interrelated beings. If patients in their daily lives do not have an adequate grasp of their disease, other healthcare professionals expect physicians to provide an explanation, not only in purely diagnostic terms but also in daily-life terms from the point of view of a person with medical authority. They also expected physicians to provide advice on the prognosis of a disease or disability from the perspective of the provision of daily-life support.

*An OT said, “I hope that physicians will play a role in listening to ideas about a more meaningful life, not just for diseases, but also with regard to background, illness, and fun.”*

*A care manager said, “If physicians can explain to patients even a little about their recovery prospects, we can form a practical vision of daily life.”*

### Personal character of open-mindedness and approachableness

Other healthcare professionals expected physicians to have an open mind, allowing them to consult other professionals proactively when confronted with complex issues. Also, they wanted to create a relationship of approachableness with physicians, which would allow them to seek their advice.

*A daily-life support coordinator said, “It is better if physicians can come down from their high mountain and be more approachable. It would be good if they can come to a forum like this.”*

*A care manager said, “I want physicians to convey a mood that makes them easy to talk to.”*

## Discussion

The role conception of physicians is as a figure of intellectual authority positioned at the top of a traditional hierarchy, with a personal character of criticism/autonomy/closedness, not accommodative of any interference from others, and upholding the Biomedical Model as an absolute standard. In response to this, the role expectation of physicians in a community is that they undertake actions that only physicians can undertake to ensure that a flat organization functions properly; that is, to liaise with other physicians, to provide medical explanations during transitions, such as when patients are transferred to different settings, and to offer healthcare support for patients who are difficult to access. This role expectation also includes a rounded perception of patients as human beings, with physicians adapting to the Bio-Psycho-Social Model, explaining to patients about their disease as an authoritative voice based on an understanding of psychosocial circumstances, and sharing the prognosis of diseases or disabilities. The personal character expected was of someone with an open mind who allows others to seek advice, as well as a sense of approachableness which makes it easier for others to seek advice.

Role conception is affected by exhibited behavior [7]. In the age when lives were lost due to infection or acute disease, medicine wielded total authority [29]. It is true that life expectancy benefitted from this and was lengthened. However, the disease model has changed alongside changes in society. Today, with the exception of cancer, lifestyle-related diseases such as cardiovascular disease, cerebrovascular disease and diabetes sit at the top of the list of causes of death [30, 31]. Especially in a community, and considering the aging of the population, intervention using the Biomedical Model alone cannot deal with frailty or associated conditions such as aspiration pneumonitis. In this context, it may be anachronistic for physicians to have absolute authority [32]. These findings may reflect the idea that physicians in the community consider themselves to be medical specialists and/or that they cling to the Biomedical Model as a normative role without finding out their role expectation. In other words, physicians are failing to verify a normative role that was originally internalized through past education and the system prevailing at that time (Fig. 1) [7, 26]. The reason that physicians criticize others is perhaps partly a reaction to their reading of these current trends, and their lack of interpersonal skills and social interaction [33].

The negative role conception of physicians held by other healthcare professionals corresponds with the role expectation they hold, namely that physicians should shift away from an unreachable top-hierarchical position and become team members of the community. For instance, physicians do not seem to listen to the opinions of other healthcare professionals, apparently since their educational or advisory position affords them few opportunities to hear such opinions [12–14]. Thus, the relationship between the role conception and the role expectation of physicians forms two sides of the same coin. Moreover, the ideal image of community physicians might be that of transformative leadership, in the context of a community-based integrated care system which has moved away from the Biomedical Model to a Bio-Psycho-Social Model [32]. Transformative leadership is principle-driven, value-based and works on relationships [34]. By directing effort into these three core elements, a person generally metamorphoses into a transformational leader [35, 36]. In a community-based integrated care system - a complex system - a well-functioning practice emerges under which independent specialties become mutually dependent [37]. To achieve this, physicians should reflect on their roles in their present community and be transformational leaders.

This study has main three limitations. First, it is limited in scope by its focus solely on the knowledge-cognitive aspects of role conception and role expectation. We hope to conduct research with future field work which focuses on cognition-behavior aspects as a means of investigating role enactment, which has an impact on clinical practice, and thereby refine the role theory for physicians in the community. Second, though participants were asked about their image and expectations of physicians in a community-based integrated care system, participants may instead have imaged the general role conception of physicians. Third, the participants in this study were healthcare professionals who might not have given their personal opinions honestly, even though the FGs were facilitated by staff in the community general support center with whom they usually interacted and the researchers were not directly involved. Any application of these findings should therefore be done with this possibility in mind. Nevertheless, by applying role theory to interpret the physicians’ role in a community setting, the present study has enriched our theoretical understanding of how different healthcare professions interpret community physicians. Among strengths, one significant finding of this study was the emergence of “role ambiguity” in a community setting, namely a discrepancy between role conception, role expectation, and normative role in “Role Theory” [9, 38, 39]. In addition, the fact that personal characteristics associated with physicians’ normative roles were extracted is unique, and has not been reported previously. Role theory suggests that role ambiguity increases an individual’s dissatisfaction with the role, hesitancy in decision making, anxiety, and confusion, and thereby leads to ineffective performance [39]. Therefore, as an implication for practice and to avoid the negative outcomes of role ambiguity, our present findings are expected to provide physicians with the opportunity to reflect on their own trustworthy behaviors in a community, not only in their relationships with patients and families but also with other healthcare professional [38, 40, 41]. Furthermore, authentic interprofessional collaboration through social interaction based on community values will prevent role ambiguity in the physician’s role in a community setting.

In summary, this study shows that what is sought from physicians working within a community-based integrated care system is that they form an open relationship with other healthcare professionals. To achieve this, physicians should consider the understanding of the role conception and role expectation that other professionals have of them, and thereby become critical of the normative role that they were exposed to and unconsciously adopted during their education within the system prevailing at that time, and flexibly adjust their role to current new environment.

## Conclusion

Healthcare professionals have the role conception that physicians who work in a community are closed authoritative figures sitting at the top of a hierarchy and wielding the Biomedical Model. Their role expectation is that they be interdependent as team members with other healthcare professionals. Physicians working in community-based integrated care systems should build open relationships with other healthcare professionals, understand their interpretative role and become role-adjusting transformative leaders.

## Data Availability

All data analyzed during the current study are available from the corresponding author on reasonable request.

## Abbreviations

FGs: Focus groups
